# Performance of nasopharyngeal swab and saliva in detecting Delta and Omicron SARS‐CoV‐2 variants

**DOI:** 10.1002/jmv.27898

**Published:** 2022-06-08

**Authors:** Tina Uršič, Rok Kogoj, Jaka Šikonja, Damijana Roškarič, Monika Jevšnik Virant, Petra Bogovič, Miroslav Petrovec

**Affiliations:** ^1^ Faculty of Medicine, Institute of Microbiology and Immunology University of Ljubljana Ljubljana Slovenia; ^2^ Department of Infectious Diseases University Clinical Centre Ljubljana Ljubljana Slovenia

**Keywords:** COVID‐19, nasopharyngeal swab, real‐time RT‐PCR, saliva, SARS‐CoV‐2 diagnostics

## Abstract

A prospective cohort study was conducted during the Delta and Omicron severe acute respiratory syndrome coronavirus type 2 (SARS‐CoV‐2) epidemic waves from paired nasopharyngeal swab (NPS or NP swab) and saliva samples taken from 624 participants. The study aimed to assess if any differences among participants from both waves could be observed and if any difference in molecular diagnostic performance could be observed among the two sample types. Samples were transported immediately to the laboratory to ensure the highest possible sample quality without any freezing and thawing steps before processing. Nucleic acids from saliva and NPS were prospectively extracted and SARS‐CoV‐2 was detected using a real‐time reverse‐transcription polymerase chain reaction. All observed results were statistically analyzed. Although the results obtained with NP and saliva agreed overall, higher viral loads were observed in NP swabs regardless of the day of specimen collection in both SARS‐CoV‐2 epidemic waves. No significant difference could be observed between the two epidemic waves characterized by Delta or Omicron SARS‐CoV‐2. To note, Delta infection resulted in higher viral loads both in NP and saliva and more symptoms, including rhinorrhea, cough, and dyspnea, whereas Omicron wave patients more frequently reported sore throat. An increase in the mean log RNA of SARS‐CoV‐2 was observed with the number of expressed symptoms in both waves, however, the difference was not significant. Data confirmed that results from saliva were concordant with those from NP swabs, although saliva proved to be a challenging sample with frequent inhibitions that required substantial retesting.

## 1 INTRODUCTION

Shortly after the severe acute respiratory syndrome coronavirus type 2 (SARS‐CoV‐2), the pandemic was declared, laboratories around the world faced unprecedented demand for molecular testing, resulting in shortages of transport medium and collection swabs as well as nucleic acid isolation and polymerase chain reaction (PCR) reagents, consumables, and even qualified medical and laboratory personnel.^1^, ^2^ As real‐time reverse‐transcription PCR (rtRT‐PCR) is considered the laboratory gold standard for SARS‐CoV‐2 detection,^3^, ^4^ clinical and reference laboratories could not simply switch to antigen detection to meet the demand for testing. Rather, rtRT‐PCR testing had to be elevated to new, higher levels of throughput and turnaround time, primarily through the use of fully integrated, automated,^5^, ^6^ and semiautomated high‐throughput systems,^7^ pooling of samples,^8^, ^9^, ^10^ logistics,^11^ and triage of samples through multiple platforms in simultaneous use.

Since the beginning of the pandemic, nasopharyngeal swab (NP swab or NPS) collected in viral transport medium has been considered the preferred specimen for molecular detection of SARS‐CoV‐2.^12^, ^13^ Although this sample is well suited for high‐precision SARS‐CoV‐2 molecular diagnostics because it allows for easy processing, low inhibition, and high detection sensitivity with a simple and rapid collection procedure, it is not without drawbacks.^14^, ^15^, ^16^, ^17^, ^18^, ^19^ First, some patients complain about the procedure and find it uncomfortable or even painful.^14^, ^15^ Second, the collection procedure cannot be standardized and therefore varies from sample to sample.^16^ Third, there have been problems due to shortages in the availability of swabs and viral transport media.^17^ Finally, new insights into the tropism of SARS‐CoV‐2 led to the testing of other samples for their putative higher suitability.^18^, ^19^

Thus, oropharyngeal swabs (OPS) and saliva have been suggested as the best samples for molecular detection of SARS‐CoV‐2,^20^ but other sample types such as alveolar lavage fluid, sputum, urine, serum/plasma, whole blood, nasal swabs (NS), corneal secretions, and even anal swabs and stool have been investigated.^21^, ^22^, ^23^ Available studies seem not to show the uniformed performance of these samples, with the varying agreement in the detection rate of SARS‐CoV‐2 RNA, but they mostly agree that the NPS is still a better sampling method choice.^12^

In September 2021, Slovenia experienced its fourth COVID‐19 wave caused by the SARS‐CoV‐2 Delta genomic variant. Delta was rapidly replaced by the Omicron genomic variant in the first 2 weeks of 2022, which was later the cause of the fifth and largest wave to date, according to a national report on the genetic variants of SARS‐CoV‐2 (data available only in the Slovenian language at: https://www.nlzoh.si/objave/sledenje-razlicicam-sars-cov-2-53/).

Although cases detected by rtRT‐PCR reached unprecedented numbers in January 2022, hospitals did not experience as great an influx of patients as during the Delta wave.

This study aimed to examine several features of SARS‐CoV‐2 infection in outpatients during the Delta and Omicron waves. Specifically, we examined the clinical relevance of saliva samples compared with NPS in general and the genomic variant. We also compared viral load in outpatients infected with the Delta and Omicron genomic variants in NPS and saliva samples and related these data to days after symptom onset, symptom severity, and vaccination status.

## 2 MATERIALS AND METHODS

### 2.1 Study design, study population, and sample collection

A head‐to‐head comparative study included individuals visiting the largest COVID‐19 swab collection center in Slovenia for routine NPS collection for SARS‐CoV‐2 testing. Written informed consent was obtained from all participants included, along with at least 1 ml of self‐collected posterior saliva sample following instructions and supervision by medical personnel onsite. In addition, a short questionnaire on clinical symptoms, such as rhinorrhea, cough, sneezing, sore throat, headache, body temperature, hoarseness, dyspnea, chest pain, duration of illness (in days), sex, age, and vaccination history. The NPS was collected in CITOSWAB VTM (nal von Minden GmbH) and paired saliva samples in an empty Saliva Collector (Biocomma Limited) without any buffer added. In the Delta wave (September–October 2021), 298 individuals, and in the Omicron wave (January 2022), 326 individuals participated. All samples were processed and analyzed immediately after collection.

### 2.2 Nucleic acid isolation and rtRT‐PCR

Total nucleic acid was isolated from 200 µl of sample mixed with 10 µl of equine arteritis virus internal control in a Nextractor NX‐48S (Genolution, Seoul, South Korea). NPS and saliva samples were processed in the same manner. Saliva was processed directly without any additives. rtRT‐PCR was performed using the CE IVD LightMix® Kit SARS‐CoV‐2 E+N UBC (TIB MolBiol) according to the manufacturer's instructions. Quantification of SARS‐CoV‐2 and human DNA was performed using gBlocks Gene Fragments (Integrated DNA Technologies) and an in‐house standard, respectively.

### 2.3 Statistical analysis

Results were collected, analyzed, and visualized in Microsoft Office 365 Excel version (Microsoft Corporation) and IBM SPSS Statistics, version 26.0 (IBM). Pearson's *χ* ^2^ test was used to compare categorical variables between waves or sample types. Numerical data were first initially tested for normality of distribution using a Shapiro–Wilk test. Normally distributed data were analyzed with the independent or paired *t*‐test. Non‐normally distributed data were compared using a nonparametric test: the Wilcoxon matched‐pairs signed‐rank test for comparing viral loads between different samples within participants and the Mann–Whitney *U* test for comparing viral loads between independent samples. A nonparametric Kruskal–Wallis *H* test was used when more than two independent groups of data were compared. A *p* ≤ 0.05 was considered statistically significant in all tests. A statistical comparison between symptomatic and asymptomatic patients included in both waves could not be performed because the total number of asymptomatic patients was too small.

## 3 RESULTS

### 3.1 Participants’ cohorts and clinical presentation

Altogether 624 outpatients with paired NPS and saliva samples participated in the study. A similar number of outpatients participated in both waves. In the Delta wave, more women were included and participants were older (Delta median of 35.5 years vs. Omicron median of 29 years); in comparison to participants from the Omicron wave, as shown in Table 1. Vaccination status was not statistically different between outpatients from both waves; however, it was observed that none of the participants in the Delta wave has already received a third vaccine dose.

**TABLE 1:** Descriptive characteristics of Delta and Omicron wave patients

Study participant characteristics	Delta wave	Omicron wave	*p*‐value (*α* = 0.05)
Female	181/298 (60.7)	169/326 (51.8)	0.025
Median age (years)	35.5	29.0	<0.001
Known vaccination status	240/298 (80.5)	309/324 (95.4)	
Vaccinated	107/240 (44.6)	155/309 (50.2)	0.729
One dose	20/240 (8.3)	17/309 (5.5)	
Two doses	87/240 (36.3)	95/309 (30.7)	
Three doses	0/240 (0.0)	43/309 (13.9)	

*Note*: For the vaccination status, values are presented as participants reporting as vaccinated with one, two, or three doses/participants with at least one vaccination dose.

Rhinorrhea, cough, and dyspnea were more frequently reported symptoms among Delta wave outpatients, whereas sore throat was more frequently reported among Omicron wave outpatients, as shown in Table 2. Participants in both waves also reported other symptoms that were not already given in the questionnaire, such as joint and/or muscle pain, diarrhea, vomiting, nausea, loss of taste or smell, and sinusitis.

**TABLE 2:** Comparison of symptoms experienced by patients from both waves

Symptoms	Delta wave	Omicron wave	*p*‐value
Symptom presence	239/249 (96)	289/312 (92.6)	0.093
Rhinorrhea	172/249 (69.1)	162/312 (51.9)	<0.001
Cough	173/249 (69.5)	182/312 (58.3)	0.007
Sneezing	112/249 (45)	123/312 (39.4)	0.185
Sore throat	103/249 (41.4)	158/312 (50.6)	0.029
Headache	161/249 (64.7)	186/312 (59.6)	0.222
Fever	101/249 (40.6)	126/312 (40.4)	0.966
Hoarseness	73/249 (29.3)	73/312 (23.4)	0.112
Dyspnea	38/249 (15.3)	28/312 (9)	0.022
Chest pain	39/249 (15.7)	32/312 (10.3)	0.056
Other symptoms	6/249 (2.4)	7/312 (2.2)	0.897

*Note*: Values are presented as participants reporting a symptom/participants with known symptoms (percent of participants with symptoms)

Other symptoms: Muscle and joint pain, nausea, vomiting, sinusitis, diarrhea, and loss of smell or taste.

### 3.2 Performance of rtRT‐PCR testing by sample type

The observed overall agreement between NPS and saliva in Delta and Omicron waves were 97% and 91.7%, respectively. In the Delta wave, saliva showed a positive agreement of 97.5% (95% confidence interval, CI: 95.1%–99.9%) and negative agreement of 96.3% (95% CI: 96.3%–93.2%) compared to NPS, whereas in the Omicron wave, saliva showed a positive agreement of 93.6% (95% CI: 90.5%–96.7%) and negative agreement of 86.7% (95% CI: 79.6%–93.7%) compared to NPS. A detailed head‐to‐head comparison between NPS and saliva from both waves is shown in Table 3. Overall, 50/624 (8%) collected saliva samples were repeated due to extraction inhibition, compared with only 3/624 (0.5%) NPS, the difference was statistically significant (*p* < 0.001). After repeated extraction and rtRT‐PCR, a valid result was obtained for all samples, thus all were included in the comparison.

**TABLE 3:** Head‐to‐head comparison of rtRT‐PCR results obtained after testing NP swab and saliva samples from Delta and Omicron wave patients

Delta wave		LightMix nasopharynx	
		POS	NEG
LightMix saliva	POS	158	4
	NEG	5	131
		163	135
*Omicron wave*			
LightMix saliva	POS	221	15
	NEG	12	78
		233	93

Abbreviations: NEG, negative; NP, nasopharyngeal; POS, positive; rtRT‐PCR, real‐time reverse‐transcription polymerase chain reaction.

### 3.3 Viral loads by sample type and genomic variant

**FIGURE 1:**
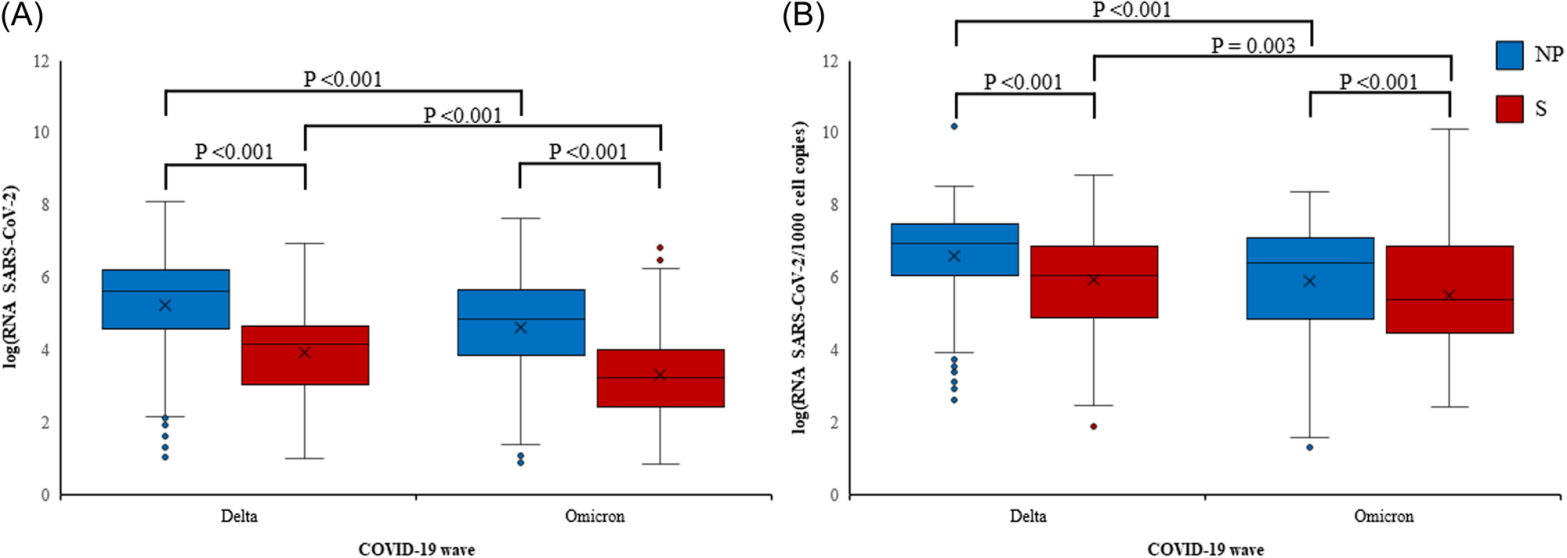
Comparison of viral loads in nasopharyngeal (NP) swabs (blue) and saliva (S) samples (red) between Delta and Omicron waves. Non‐normalized (A) and normalized (B) viral loads are presented as log_10_ (SARS‐CoV‐2 RNA copies/µl) or as log_10_ (SARS‐CoV‐2 RNA copies/1000 cell copies), respectively. The box represents the first and third quartile, the line in the box, the median, the cross, the mean value, and the whiskers, the minimal and maximal value, excluding outliers that are presented as individual dots. Outliers are defined as values that deviate >1.5 times the interquartile range from the box limits. Statistical significance for individual comparisons is shown as *p*‐values. SARS‐CoV‐2, severe acute respiratory syndrome coronavirus 2.

Both Delta and Omicron wave outpatients had statistically significantly higher viral loads (*p* < 0.001) in NPS compared to saliva. A mean of 5.08 log_10_ RNA copies/µl (Delta wave) and 4.56 log_10_ RNA copies/µl (Omicron wave) was detected in NPS compared with a mean of 3.85 log_10_ RNA copies/µl (Delta wave) and 3.26 log_10_ RNA copies/µl (Omicron wave) in saliva. A similar statistically significant difference was observed when comparing the same sample types (NPS vs. saliva) between waves. Statistically, significantly higher mean viral loads were observed in NPS (*p* < 0.001) and saliva (*p* < 0.001) in Delta wave compared to Omicron wave outpatients. The results are shown in Figure 1A.

When the SARS‐CoV‐2 viral load was normalized on the human reference gene (RNA SARS‐CoV‐2/1000 cell copies) still Delta and Omicron wave outpatients had statistically significantly higher mean RNA SARS CoV‐2/1000 cell copies in NPS (*p* < 0.001) compared to saliva. A mean of 6.58 log_10_ RNA SARS‐CoV‐2/1000 cell copies (Delta wave) and 5.89 log_10_ RNA SASR‐CoV‐2/1000 cell copies (Omicron wave) was detected in NPS compared with a mean of 5.92 log_10_ RNA SARS‐CoV‐2/1000 cell copies (Delta wave) and 5.5 log_10_ RNA SARS‐CoV‐2/1000 cell copies (Omicron wave) in saliva. A similar statistically significant difference was observed when comparing the same sample types (NPS vs. saliva) between waves. Statistically, significantly higher mean viral loads were observed in NPS (*p* < 0.001) and saliva (*p* = 0.003) in Delta wave compared to Omicron wave outpatients. The results are shown in Figure 1B.

### 3.4 Viral load temporal dynamics by sample type and genomic variant

**FIGURE 2:**
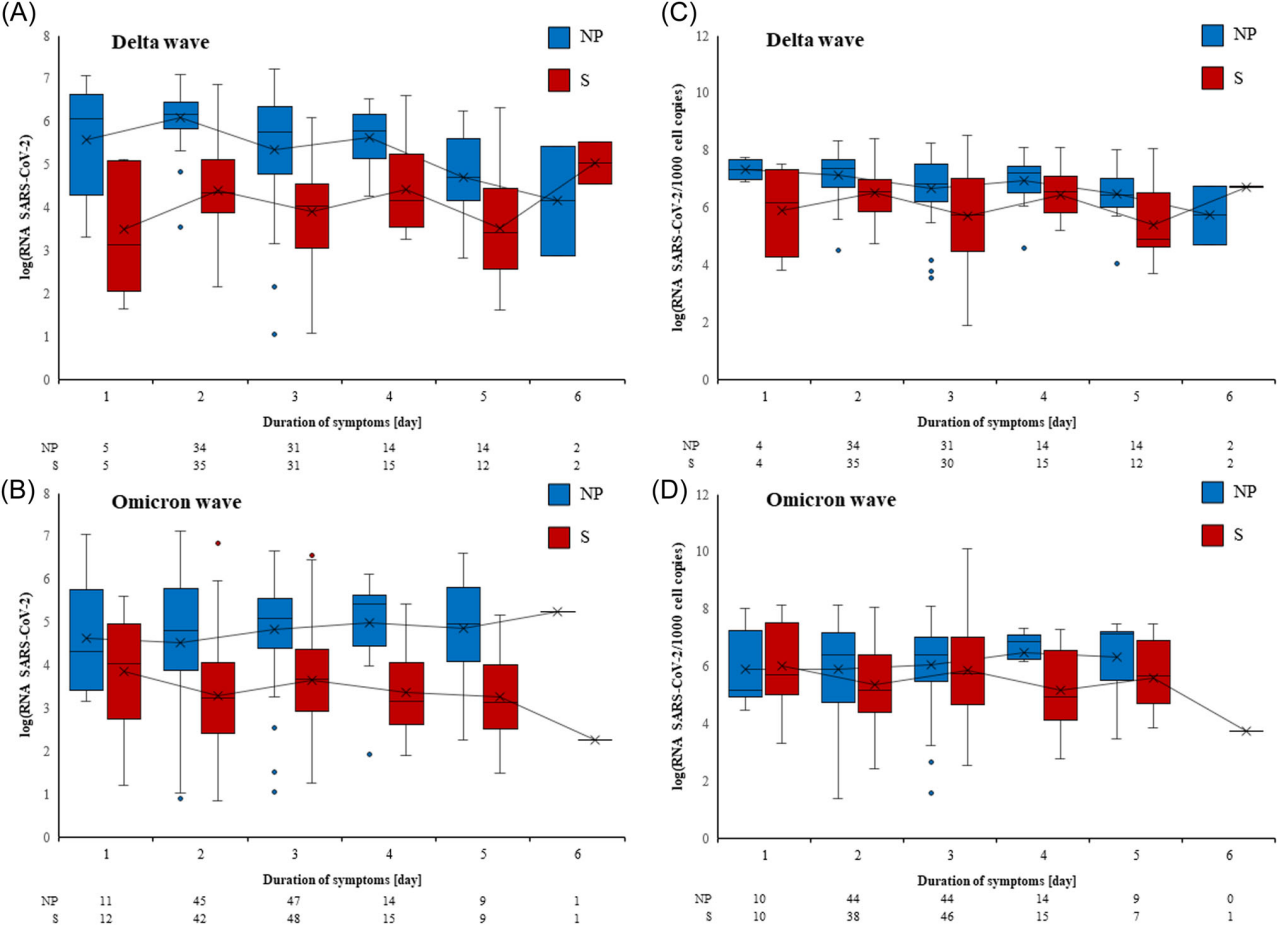
Viral loads at respective days after symptom onset in Delta (A and C) and Omicron (B and D) wave patients. Nasopharynx (nasopharyngeal, NP) samples are presented in blue and saliva (S) samples in red. Non‐normalized (A and B) and normalized (C and D) viral loads are presented as log_10_ (SARS‐CoV‐2 RNA copies/µl) or as log_10_ (SARS‐CoV‐2 RNA copies/1000 cell copies), respectively. The box represents the first and third quartile, the line in the box the median, the cross the mean value, and the whiskers the minimal and maximal value, excluding outliers that are presented as individual dots. Outliers are defined as values that deviate >1.5 times the interquartile range from the box limits. A trend line connects the means of viral loads across days of symptom duration. The number of participants on respective days after symptom onset included in calculations is presented below each graph. SARS‐CoV‐2, severe acute respiratory syndrome coronavirus 2.

The dynamics of log_10_ RNA copies/µl were assessed in both sample types for Delta and Omicron wave outpatients at 1, 2, 3, 4, 5, and 6 days after symptom onset. The number of SARS‐CoV‐2‐positive outpatients whose samples were collected later than 6 days after symptom onset was small (10 in the Delta wave and 3 in the Omicron wave) and they were therefore excluded from further analysis. The results confirm that mean log_10_ RNA copies/µl were consistently higher in NPS compared with saliva in both waves, regardless of the day after symptom onset. This pattern was less pronounced only in outpatients in the Omicron wave whose samples were collected 1 day after symptom onset. In this group, mean log_10_ RNA copies/µl in saliva approached the viral load in NPS but decreased more rapidly in subsequent days compared with the mean log_10_ RNA copies/µl in NPS, as shown in Figure 2. Compensation for the amount of human DNA in the sample (normalization) did not affect the overall pattern, as shown in Figure 2C,D. When the viral load was normalized in outpatients in the Omicron wave whose samples were collected 1 day after symptom onset, in this group, the mean log_10_ RNA SARS‐CoV‐2/1000 cell copies in saliva was higher than the mean log_10_ RNA SARS‐CoV‐2/1000 cell copies in NPS. However, the viral load in saliva decreased more rapidly in subsequent days compared with mean log_10_ RNA SARS‐CoV‐2/1000 cell copies in NPS, as shown in Figure 2D.

### 3.5 Viral loads and symptom presentation by sample type, genomic variant, and vaccination status

Delta wave outpatients reported significantly more symptoms than Omicron wave outpatients (*p* = 0.005). Although the mean log RNA copies/µl in NPS and saliva samples (in both the Delta and Omicron waves) increased with the number of symptoms expressed (Groups: 0–2 symptoms, 3–5 symptoms, and ≥6 symptoms), the difference was not statistically significant, as shown in Table 4.

**TABLE 4:** Mean log RNA copies/µl in NPS and saliva samples according to the number of symptoms reported in the Delta and Omicron waves

Wave	No. of symptoms	NP mean log RNA	S mean log RNA	*p*‐value NP	*p*‐value S
Delta	0–2	5.16	3.77	0.635	0.431
	3–5	5.32	3.91		
	≥6	5.40	4.20		
Omicron	0–2	4.40	3.13	0.052	0.081
	3–5	4.69	3.32		
	≥6	4.93	3.60		

*Note*: The Kruskal‐Wallis H test was used to compare viral loads across symptom groups individually for sample type and epidemic wave.

Abbreviations: NP, nasopharyngeal; NPS, nasopharyngeal swab; S, saliva.

When looking for correlations between viral load and the specific symptom, statistically significantly higher log_10_ RNA copies/µl were observed only in NPS for outpatients that reported rhinorrhea compared with outpatients that did not report this symptom (*p* = 0.05) in the Delta wave. A correlation between viral load and specific symptoms was not observed in any other combination, including saliva samples from participants in the Omicron wave that reported sore throat (*p* = 0.081).

A comparison of vaccinated and unvaccinated participants in both waves showed no difference in NPS or saliva log RNA copies/µl. Also, no difference was observed in the occurrence of symptoms between the two groups (*p* = 0.483). No statistical analysis was performed between symptomatic and asymptomatic participants because only a very small number of asymptomatic participants were included in the study (23/624).

In 13/298 saliva samples in Delta wave and 37/326 saliva samples in Omicron wave, inhibition of nucleic acids extraction was observed. On the other side, inhibition of nucleic acid extraction in only 3/298 NPS from the Delta and 0/326 from the Omicron wave was observed. Altogether, 8% of saliva samples (50/624) and only 0.5% of NPS (3/624) had to be re‐extracted due to insufficient quality of primary nucleic acid extraction, with the difference being statistically significant between the sample types (*p* < 0.001).

## 4 DISCUSSION

The results of this prospective head‐to‐head cohort study on 624 paired NPS and saliva samples from outpatients during the Delta and Omicron waves indicate more severe clinical presentation in participants infected with the Delta SARS‐CoV‐2 genomic variant than in Omicron. The data show that Delta wave outpatients overall reported statistically significantly more symptoms than Omicron wave outpatients. Delta outpatients more frequently reported rhinorrhea, cough, and dyspnea compared to Omicron outpatients, who more frequently reported sore throat. This observation is in line with some other studies reporting more severe clinical presentation in Delta variant infections compared to the Omicron variant of SARS‐CoV‐2.^24^, ^25^, ^26^, ^27^, ^28^ This study also reveals that Delta wave patients had significantly higher log RNA copies/µl in NPS and saliva compared to Omicron wave patients. Genomic variant‐dependent NPS viral load observation is not unexpected; Migueres et al.^28^ showed higher viral loads in patients NPS infected with Delta compared to the Alpha variant, independent of patients’ age, sex, symptoms, and vaccination status. Interestingly, Salvagno et al.^29^ showed higher NPS viral loads in patients infected with the Omicron variant compared to patients infected with the Alpha variant. Moreover, a study by Fall et al.^30^ shows no significant difference in cycle threshold values from upper respiratory samples between Delta and Omicron genomic variant‐infected patients. Such reports point to an inconclusive verdict on whether SARS‐CoV‐2 viral loads are truly connected to virus genomic variants or another variable. Most studies do not take into account normalization for human DNA as a sample quality/quantity surrogate. However, in this study, normalization did not affect the final result, except for higher mean log_10_ RNA SARS‐CoV‐2/1000 cell copies in Omicron outpatients in saliva compared to NPS. In our opinion, lower viral loads for the Omicron genomic variant are more in line with clinical observations such as those by Iuliano et al.^26^ which showed that patients infected with the Omicron variant have shorter hospital stays and less frequent intensive care unit admissions compared to patients infected with the Delta variant, which further seems to be in concordance with faster virus clearance. These findings could also be explained by evidence of less efficient replication and fusion activity of Omicron when compared with Delta variant in TMPRSS2‐expressed cells, which could be the reason for less severe lung pathology.^26^, ^31^

Although the results between NPS and saliva in the Delta wave were highly concordant, we observed lower overall agreement between the NPS and saliva in Omicron wave outpatients than in Delta wave outpatients. Lower concordance among sample NPS and saliva in the Omicron wave could be the result of the several mutations in spike protein and their contribution to a viral escape antibody response.^32^ The Omicron variant replicates less efficiently in lung epithelial cells compared with the Delta variant, thus further could contribute to increased transmissibility of Omicron, as well as its apparent reduced disease severity. The studies show that Omicron prefers endosomal fusion to cell‐surface fusion, but, its wider ability to infect different cell types makes the Omicron variant more transmissible.^32^, ^33^

Another interesting finding of our study is that log RNA of SARS‐CoV‐2 in both waves was significantly higher in NPS compared with saliva samples, independent of the day of sample collection after symptoms onset. This finding supports the primary knowledge about the quality and suitability of NPS as the optimal sample of choice for the detection of not only SARS‐CoV‐2 but also other respiratory viruses and is in concordance with WHO and CDC instructions and recommendations.^34^, ^35^ Our results support the findings of a meta‐analysis by Lee et al.^12^ in which saliva, NS, and OPS captured a lower percentage of positives than NPS, whereas combined oropharyngeal/NS matched NPS performance. In Callahan et al.,^36^ on 385 paired NPS and saliva samples, and in Escobar et al.,^37^ on 127 paired samples, excellent concordances between the two samples were observed although the viral load of SARS‐CoV‐2 in both studies was lower in saliva samples compared to NPS samples. However, Beyene et al.^20^ found saliva to be not only a good alternative sample for SARS‐CoV‐2 diagnostics but superior to NPS in patients on the day of hospital admission.

Based on our observations, NPS remains a better choice for the detection of SARS‐CoV‐2 RNA for both Delta and Omicron genomic variants. It might be that the use of saliva instead of NPS offers some advantages at first glance; similar detection performance as NPS, less patient discomfort, no obligate need for monitoring by medical personnel, and even collection at home.

However, we believe that consideration should be given to an important aspect that is often overlooked in evaluation studies. The complex matrix of saliva poses a serious limitation to its usability, due to its viscosity and the presence of inhibitors. Consequently, saliva sample processing may lead to increased retesting, which, in turn, affects the workflow of already overburdened diagnostic laboratories and prolong time‐to‐result significantly. In this prospective head‐to‐head cohort study, a total of 8% of saliva samples were inhibited and required repeat testing, compared with 0.5% of NP swabs, with the difference being statistically significant. There is reason to believe that the inhibition rate could be even higher if saliva samples were not collected under the supervision of healthcare professionals. In addition, some elderly patients in this study found saliva collection difficult and far more frustrating than NP swab collection as a collection of saliva could take even up to 10 min. Some reported that delivering a sufficient amount of saliva was challenging, which was even more pronounced in patients with neurologic impairment or orofacial dyskinesia.^15^

Several strengths of the study can be highlighted. Comparable numbers of patients from the Delta and Omicron waves were prospectively enrolled. Paired NP swab/saliva samples from each patient were collected at the same time and nucleic acid isolation and rtRT‐PCR from both samples were performed on the same day. Therefore, no prolonged storing or freezing/thawing occurred for any of the samples. The saliva samples were collected under the supervision of healthcare personnel, thus excluding inhibitions of saliva samples due to incorrect collection procedures and eliminating the possibility of saliva samples of inadequate volume. On the other hand, one limitation of the study is not determining the SARS‐CoV‐2 genetic variant present in the samples directly, but rather taking into account the results from national SARS‐CoV‐2 variant screening, which reported 100% Delta and Omicron during the first and second parts of the study, respectively. Another limitation could be the nonuse of a mucolytic agent in the processing of saliva samples, and thus in some cases, nonhomogeneous dispersion of viral particles might have occurred in samples due to saliva consistency. However, not using a mucolytic agent, on the other hand, means that saliva samples were not diluted and thus the viral load in the saliva samples was not affected.

## 5 CONCLUSION

Although saliva showed very good concordance with NPS, the viral load of SARS‐CoV‐2 in both waves was significantly higher in NPS independent of the day of sample collection. Moreover, statistically, significantly more inhibitions of saliva samples were observed compared to NPS. From the result concordance point of view, we propose an OPS instead of saliva in diagnostics of SARS‐CoV‐2 when the NPS is harder to collect, but when possible, combined NPS/OPS samples would probably be the best option. This would probably avoid most of the discordant results with a maximal increase of the sensitivity and limiting the number of inhibited samples.

## AUTHOR CONTRIBUTIONS

The manuscript has been read and approved by all authors and was not submitted, published, and accepted for publication elsewhere. Tina Uršič and Miroslav Petrovec designed and planned the study. Tina Uršič and Jaka Šikonja wrote the protocol. Tina Uršič, Jaka Šikonja, and Damijana Roškarič were responsible for project execution and sample collection. Tina Uršič, Jaka Šikonja, Damijana Roškarič, and Monika Jevšnik Virant conducted the laboratory analysis. Jaka Šikonja and Petra Bogovič were responsible for the written consent of the participants included in the study. Tina Uršič, Jaka Šikonja, Rok Kogoj, and Monika Jevšnik Virant conducted the primary data analysis and drafted the manuscript. Miroslav Petrovec and Petra Bogovič supervised the manuscript draft. All authors have read and agreed to the published version of the manuscript.

## CONFLICT OF INTEREST

The authors declare no conflict of interest.

## ETHICS STATEMENT

This study was approved by the National Medical Ethics Committee of Slovenia (No. 0120‐211/2020/25).

## Data Availability

This manuscript has no associated data to make it available.
